# Susceptibility of CoFeB/AlO*_x_*/Co Magnetic Tunnel Junctions to Low-Frequency Alternating Current

**DOI:** 10.3390/nano3040574

**Published:** 2013-10-15

**Authors:** Yuan-Tsung Chen, Zu-Gao Chang

**Affiliations:** Department of Materials Science and Engineering, I-Shou University, Kaohsiung 840, Taiwan; E-Mail: isu10107014m@cloud.isu.edu.tw

**Keywords:** magnetic tunnel junctions (MTJs), indirect exchange coupling, low-frequency alternate-current magnetic susceptibility (χ_ac_), resonance frequency (*f*_res_)

## Abstract

This investigation studies CoFeB/AlO*_x_*/Co magnetic tunneling junction (MTJ) in the magnetic field of a low-frequency alternating current, for various thicknesses of the barrier layer AlO*_x_*. The low-frequency alternate-current magnetic susceptibility (χ_ac_) and phase angle (θ) of the CoFeB/AlO*_x_*/Co MTJ are determined using an χ_ac_ analyzer. The driving frequency ranges from 10 to 25,000 Hz. These multilayered MTJs are deposited on a silicon substrate using a DC and RF magnetron sputtering system. Barrier layer thicknesses are 22, 26, and 30 Å. The X-ray diffraction patterns (XRD) include a main peak at 2θ = 44.7° from hexagonal close-packed (HCP) Co with a highly (0002) textured structure, with AlO*_x_* and CoFeB as amorphous phases. The full width at half maximum (FWHM) of the Co(0002) peak, decreases as the AlO*_x_* thickness increases; revealing that the Co layer becomes more crystalline with increasing thickness. χ_ac_ result demonstrates that the optimal resonance frequency (*f*_res_) that maximizes the χ_ac_ value is 500 Hz. As the frequency increases to 1000 Hz, the susceptibility decreases rapidly. However, when the frequency increases over 1000 Hz, the susceptibility sharply declines, and almost closes to zero. The experimental results reveal that the mean optimal susceptibility is 1.87 at an AlO*_x_* barrier layer thickness of 30 Å because the Co(0002) texture induces magneto-anisotropy, which improves the indirect CoFeB and Co spin exchange-coupling strength and the χ_ac_ value. The results concerning magnetism indicate that the magnetic characteristics are related to the crystallinity of Co.

## 1. Introduction

Recently, ferromagnetic exchange-coupling in magnetic fields has been extensively examined. Exchange-coupling has been discussed following the discovery of spintronics [1,2,3]. Magnetic tunneling junctions (MTJs) have a sandwiched structure, which is composed of a top ferromagnetic (FM1) layer, an insulating tunneling layer (spacer), and a bottom ferromagnetic (FM2) layer, which can be utilized in high-density read/write heads, magnetoresistance random access memories (MRAM) and gauge sensor applications. This structure yields a very large magnetoresistance (MR) owing to spin-dependent tunneling effect [4,5,6,7,8,9,10]. Numerous factors influence the magnetoresistance, such as the indirect spin exchange-coupling of ferromagnetic layers, and the quality of the insulating tunneling layer, which affect the magnetic performance. The indirect exchange coupling between FM1 and FM2 layers in MTJs is interesting to investigate because the indirect exchange coupling can influence saturation magnetization (*M*_s_) and coercivity (*H*_c_) [11]. However, a high-quality MTJ must have superior ferromagnetic layers, a high spin-polarization, a microstructure that is as close to ideal as possible, and indirect spin exchange-coupling between the FM1 and FM2 ferromagnetic layers. However, most MTJ research has focused on tunneling magnetoresistance (TMR). Additionally, most relevant investigations of magnetism have focused on high-frequency magnetic impedance (MI) [12]. Only a few have focused on low-frequency alternate-current magnetic susceptibility (χ_ac_) and the optimal resonance frequency (*f*_res_). Susceptibility to low-frequency alternate-current magnetism and the effect of the thickness of the tunneling barrier on indirect exchange coupling between FM1 and FM2 in the CoFeB/AlO*_x_*/Co junction are worthy of study. In this study, the thickness of CoFeB and Co is 75 Å and the AlO*_x_* barrier thickness is varied among 22, 26, and 30 Å. The X-ray diffraction patterns (XRD) includes a hexagonal close-packed (HCP) Co(0002)-textured structure at 2θ = 44.7°, and AlO*_x_* and CoFeB are amorphous phases. The results concerning χ_ac_ reveal that the susceptibility distribution is optimal at low frequency. The three highest values of susceptibility for each sample are averaged. When the thickness of the AlO*_x_* barrier is 30 Å, the mean susceptibility is highest, at 1.87, it is lowest, 1.19, at a barrier thickness of 22 Å. In summary, the susceptibility was very sensitive to variable AlO*_x_* barrier thickness. The susceptibility results demonstrate that the magnetic characteristics are related to the Co crystallinity and the thickness of the AlO*_x_* barriers.

## 2. Results and Discussion

**Figure 1:**
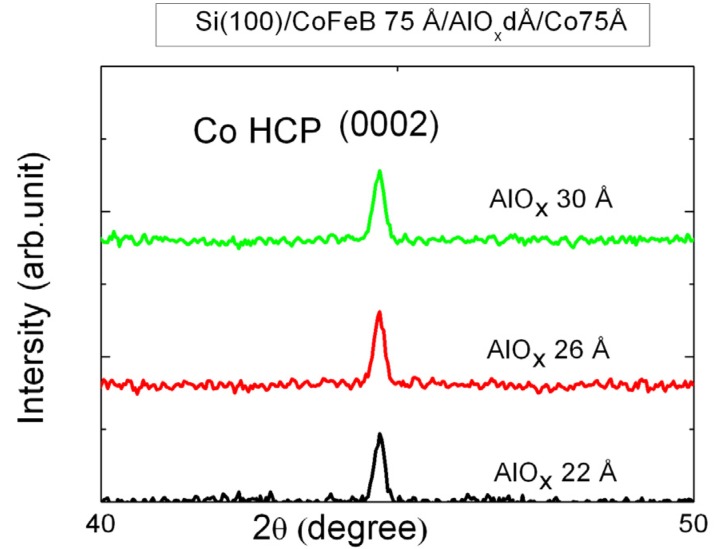
X-ray diffraction patterns of CoFeB(75 Å)/AlO*_x_*(*d* Å)/Co(75 Å) magnetic tunneling junction (MTJ).

Figure 1 presents the XRD patterns of the laminated CoFeB(75 Å)/AlO*_x_*(*d*)/Co(75 Å) MTJ junctions. They reveal that the junctions have a hexagonal close-packed (HCP) Co(0002) texture at 2θ = 44.7°. The microstructures of CoFeB and AlO*_x_* are amorphous. According to previous references [13,14], we can also reasonably conclude that the diffracted peak is Co(0002). Therefore, the HCP Co(0002) was identified herein.

**Figure 2:**
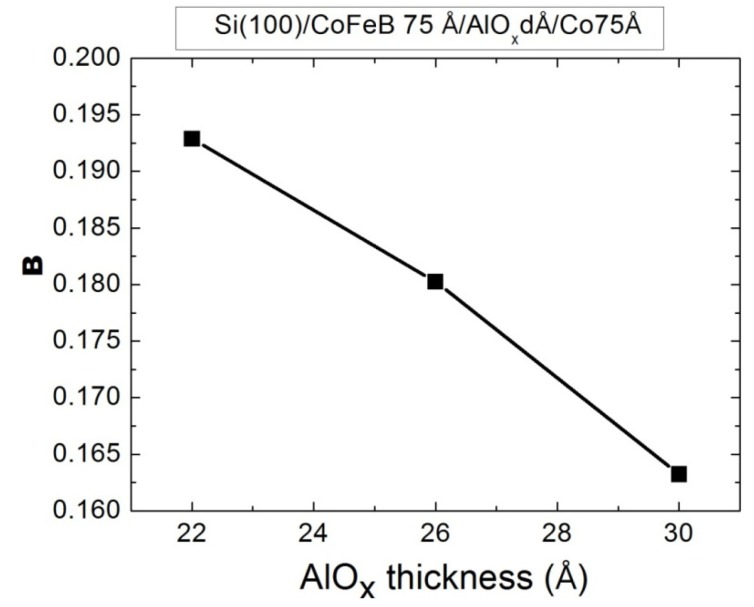
Full width at half maximum (FWHM) (*B*) as a function of thickness (*d*) of AlO*_x_* layer.

The full width at half maximum (FWHM, *B*) of the Co(0002) peak as a function of AlO*_x_* thickness was extracted from a mathematical fit to the data, which is shown in Figure 2. Scherrer’s formula can be written as:

*D* = 0.9λ/*B* cosθ (1)

where *D* represents the grain size; λ is the wavelength of the CuK_α1_ line; *B* is the full width at half maximum of the (0002) peak and θ is the half angle of the diffraction peak. This formula can be applied to calculate the grain size and determine the crystallinity [15]. From Figure 2, *B* is reduced by increasing the AlO*_x_* tunnel barrier thickness, reducing the FWHM of the peak of the Co layer. This phenomenon explains that the small *B* can induce the increase in the grain size of Co and the enhancement of the Co(0002) texture. In the deposited structure, the thickness and crystallinity of seed layer play an important parameter to providing the crystallinity of top layer [16]. In this study, it suggests that the AlO*_x_* thickness is related to grain size and crystallinity of Co layer. Moreover, the related study also indicates that the AlO*_x_* thickness of MTJ can affect Co structure, grain size, and exchange coupling strength [11]. The magneto crystalline anisotropy of Co(0002) texture can enhance its magnetic properties [17].

**Figure 3:**
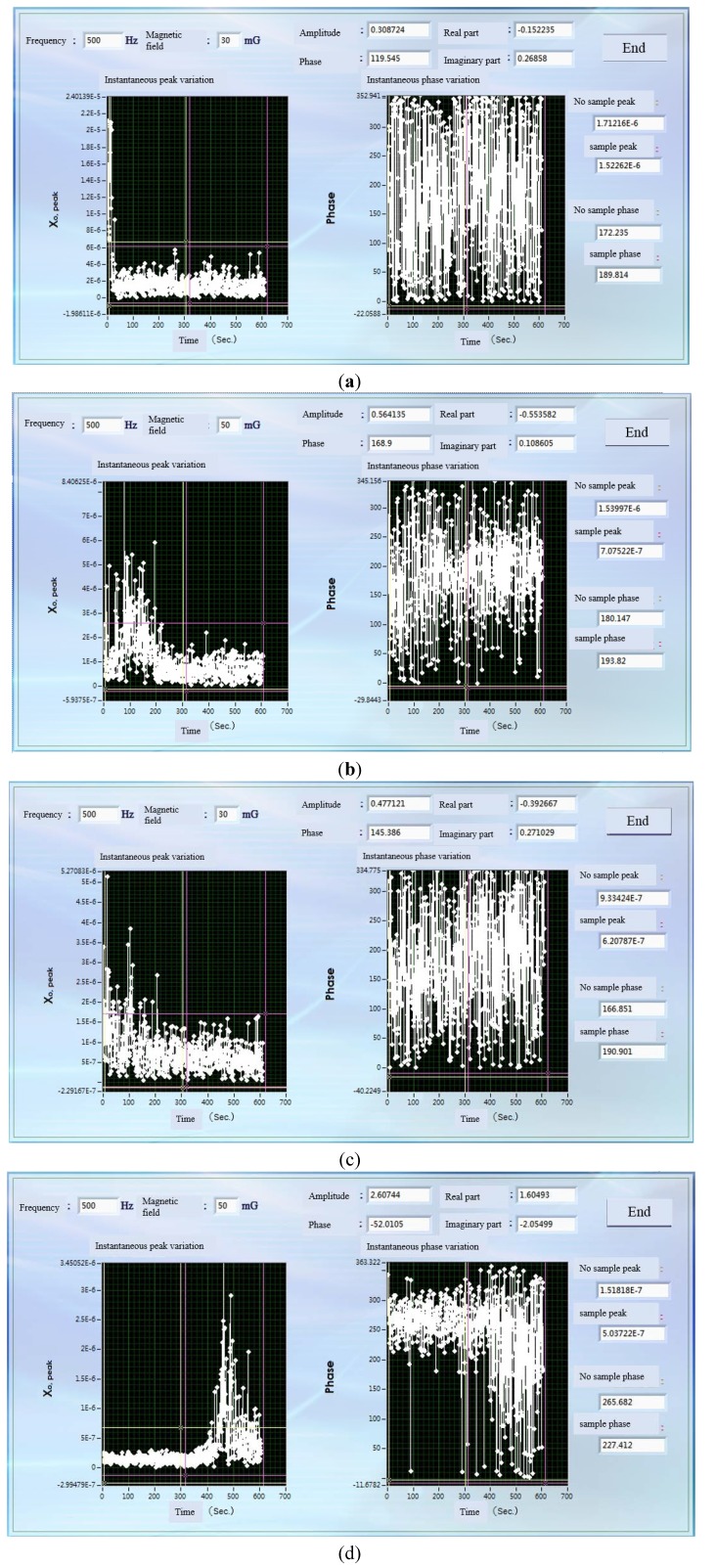
(**a**) The perpendicular χ_ac_signal of MTJ sample when the external field is 30 mOe; (**b**) The perpendicular χ_ac_signal of MTJ sample when the external field is 50 mOe; (**c**) The in-plane χ_ac_signal of MTJ sample when the external field is 30 mOe; (**d**) The in-plane χ_ac_signal of MTJ sample when the external field is 50 mOe.

Figure 3 shows the different magnitude and direction of the external magnetic field are provided in χ_ac_ measurement. According to the result, the in-plane χ_ac_signal of MTJ is larger than the perpendicular χ_ac_signal. It indicates that in-plane direction is easy-axis magnetization of MTJ.

**Figure 4:**
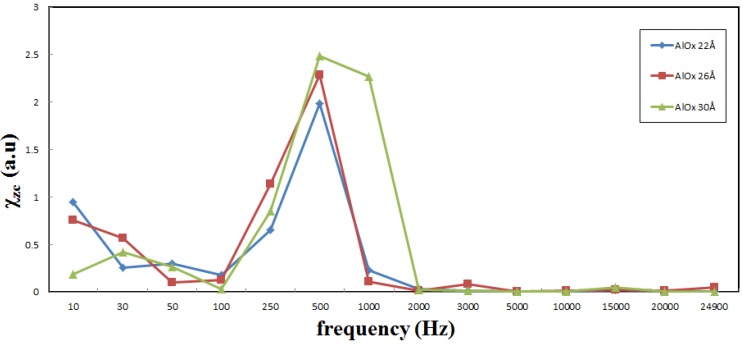
Susceptibility (χ_ac_) of CoFeB/AlO*_x_* (*d* = 22, 26 and 30 Å)/Co MTJs as a function of frequency, 10–25,000 Hz.

Figure 4 shows the low-frequency alternate-current magnetic susceptibility (χ_ac_) of the multilayered CoFeB/AlO*_x_*/Co with AlO*_x_* barrier thicknesses of 22, 26 and 30 Å and a variable frequency from 10 to 25,000 Hz. The χ_ac_ has the following physical meaning. The magnetic material under the external AC magnetic field shows a magnetic property called multiple-frequency AC magnetic susceptibility χ_ac_. The origin of χ_ac_ is due to the association between magnetic spin interactions. The frequency of the applied AC magnetic field equals the frequency of oscillation of the magnetic dipole. Hence, the frequency of the peak of the low-frequency magnetic susceptibility corresponds to the resonant frequency of the oscillation of the magnetic dipole moment inside domains. The χ_ac_ peak indicates the spin exchange-coupling interaction and dipole moment of domain under frequency [18]. It is reasonably concluded that the physical meaning peaks of the low frequency susceptibility indicate the magnetic exchange coupling between CoFeB and Co layers. The susceptibility peaks relate to the exchange interaction between CoFeB and Co layers closely. The high χ_ac_ peaks are corresponding to high exchange coupling. From Figure 4, the optimal susceptibility was obtained at the optimal resonance frequency (*f*_res_) of 500 Hz for independently of AlO*_x_* barrier thickness. It indicates that the optimal exchange-coupling strength of MTJ occurs at 500 Hz. The maximum χ_ac_ corresponds to the maximum spin sensitivity at *f*_res_. Additionally, the susceptibility of the films sharply fell to zero as the frequency was increased over 1000 Hz. The MTJ with a AlO*_x_* thickness of 30 Å had the highest χ_ac_ of approximately 1.87 at a *f*_res_ of 500 Hz because Co(0002) texture induces a magneto nanocrystalline anisotropy with the maximum χ_ac_ and *f*_res_ effect [17]. From this result, multilayered MTJs are reasonably inferred to be ideal for use in low-frequency sensors and transformers. Moreover, for mechanical components, the optimal condition is suitable in each MTJ, because the optimal resonance frequency of 500 Hz is stable.

**Figure 5:**
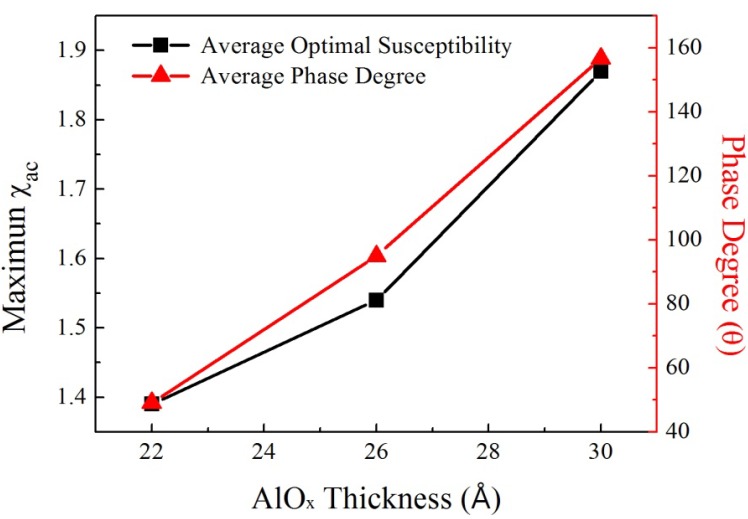
Mean optimal susceptibility and average phase angle as functions of AlO*_x_* barrier thickness.

Figure 5 plots the mean optimal susceptibility and phase angle for AlO*_x_* barriers with thicknesses of 22, 26, and 30 Å. The average phase angle corresponds to the mean optimal susceptibility. It is 49.07°, 94.94°, and 156.71°, at thicknesses of 22, 26, and 30 Å, respectively. From Table 1 and Figure 5, the mean optimal susceptibility increases from 1.19 to 1.87 as the average phase angle varies form 49.07° to 156.71°. The mean maximum χ_ac_ and phase angle increase with the AlO*_x_* barrier thickness because the magneto-anisotropy of Co(0002) texture induces strong indirect spin exchange coupling between CoFeB and Co, increasing χ_ac_. The results in Figure 5 also indicate that χ_ac_ increases with the phase angle. The χ_ac_ and the phase angle follow vary similarly. Moreover, an increasing phase angle is associated with increasingly sensitive to spin exchange-coupling strength, which is observed as a high susceptibility.

**Table 1:** High optimal susceptibility, average optimal susceptibility, and resonance frequency of maximum mean susceptibility.

*d* (Å)	Optimal susceptibility No. 1	Optimal susceptibility No. 2	Optimal susceptibility No. 3	Average optimal susceptibility	Optimal resonance frequency
22	1.9861	0.9452	0.6536	1.1950	500 Hz
26	2.2841	1.1405	0.7594	1.3947	500 Hz
30	2.4905	2.2711	0.8487	1.8700	500 Hz

## 3. Experimental Section

A multilayered MTJ was deposited on a Si(100) substrate by DC and RF magnetron sputtering. The typical base chamber pressure was less than 2 × 10^−7^ Torr, and the Ar working chamber pressure was 5 × 10^−3^ Torr. The MTJs had the structure Si(100)/CoFeB(75 Å)/AlO*_x_*(*d*)/Co(75 Å) with *d* = 22, 26 and 30 Å. The target composition of the CoFeB alloy was 40 at.% Co, 40 at.% Fe, and 20 at.% B. To form an AlO*_x_* barrier, Al was firstly deposited on the bottom FM electrode CoFeB layer, and the AlO*_x_* layer was then formed by reactive sputtering in an oxidizing atmosphere that comprised a mixture of Ar/O_2_ in the ratio 9:16. The plasma oxidation time varied from 50 to 70 s as the initial thickness of the Al layer increased from 22 to 30 Å. To examine the microstructure, the degree of Co(0002) layer texturing was characterized by X-ray diffraction (XRD) using CuK_α1_ radiation. The in-plane low-frequency alternate-current magnetic susceptibility (χ_ac_) of MTJ was investigated using an χ_ac_ analyzer (XacQuan, MagQu Co. Ltd., Sindian City, Taiwan). About the χ_ac_ measurement, the referenced standard sample is calibrated by χ_ac_ analyzer with an external field. The size of standard sample is 5 mm × 5 mm. After the calibration is finish, the same size MTJ is placed in χ_ac_ measurement. The driving frequency ranged from 10 to 25,000 Hz. The χ_ac_ is determined through the magnetization measurement. All measured samples had the same shape and size to eliminate the demagnetization factor. The χ_ac_ valve is unitless, because the χ_ac_ result is corresponding to referenced standard sample.

## 4. Conclusions

The CoFeB/AlO*_x_*/Co multilayer film is used to investigate the strength of indirect spin exchange-coupling between CoFeB and Co strength and the magnetic property χ_ac_, in a low-frequency alternating magnetic field, for various thicknesses of the barrier layer AlO*_x_*. The X-ray diffraction patterns include a main peak from highly (0002)-textured hexagonal close-packed (HCP) Co; AlO*_x_* and CoFeB are amorphous phases. The full width at half maximum (FWHM) of the Co(0002) peak decreases as the AlO*_x_* thickness increases, indicating that the Co layer becomes more crystalline. The results concerning the magnetism of all film samples demonstrate that the maximum χ_ac_ value is obtained at the optimal *f*_res_ of 500 Hz. As the frequency increased further to 1000 Hz, the susceptibility rapidly declined. Beyond 1000 Hz, it decreased sharply, almost to zero. As the barrier layer thickness increased, the χ_ac_ and phase angle increased because the magneto-anisotropy of the Co(0002) texture induced strong indirect spin exchange coupling between CoFeB and Co, increasing χ_ac_. Finally, the average optimal susceptibility of CoFeB(75 Å)/AlO*_x_*(30 Å)/Co(75 Å) reached 1.87. The susceptibility results indicate that the magnetic characteristics are related to the Co crystallinity and the thickness of the AlO*_x_* barrier. These multilayered MTJs have a high susceptibility in a low-frequency alternate-current magnetic field, making them favorable for use in low-frequency storage drives and magnetic recording media.
